# Resilience and Aging Among Black Gay and Bisexual Older Men

**DOI:** 10.3390/ijerph22081226

**Published:** 2025-08-06

**Authors:** Angela K. Perone, Beth Glover Reed, Larry M. Gant

**Affiliations:** 1 School of Social Welfare, University of California, Berkeley, CA 94720, USA; 2 School of Social Work, University of Michigan, Ann Arbor, MI 48109, USA; bgr@umich.edu (B.G.R.); lmgant@umich.edu (L.M.G.)

**Keywords:** intersectionality, sexual orientation, race, critical consciousness, health inequities, health disparities, social determinants of health

## Abstract

Black gay and bisexual older men face numerous barriers across the life course that can contribute to negative health and well-being as they age. Drawing on strengths-based social determinants discussed in the health literature and literature on intersectionality, justice, and critical consciousness, this study examines qualitative data from seventeen Black gay and bisexual older men about sources and strategies of resilience and thriving amidst intersecting systems of power and oppression that shape health inequities. The findings revealed an evolution of positive support networks across their life courses, including biological family and families of choice such as “houses” and support groups. Early and ongoing negative experiences relating to intersecting positionalities (e.g., race, gender, sexual orientation) also provided sources of strength and resilience. Participants identified three strategies for building resilience and thriving: naming external ignorance, acknowledging common struggles, and reconciling contradictions. These strategies reflected various levels of critical consciousness that helped them navigate complex and intersecting systems of power that they encountered as Black gay men across the life course. Overall, the findings underscore the importance of considering intersecting systems of power and critical consciousness when examining resilience and social determinants of health and contribute new insights on a vastly understudied population.

## 1. Introduction

Research has tended to emphasize what several scholars have recently referred to as “vulnerability-focused conceptualizations” of social determinants of health [1,2,3,4] that examine structural disadvantages as opposed to protective measures and what communities do well amidst structural inequities. Social determinants of health comprise “the conditions in which people are born, grow, work, live, and age and the wider set of forces and systems shaping the conditions of daily life” [5]. This growing body of literature underscores the importance of addressing both harmful impacts of destructive social determinants of health as well as mechanisms and strategies that mitigate or help protect communities in the face of such structural inequities.

Resilience, which often focuses on overcoming adversity, provides an important shift from risk and vulnerability to strength and capacity and offers promising opportunities for studying complex questions about well-being [1,6]. Using Collins and Bilge’s [7] intersectionality framework of power, this article builds on theoretical and empirical research on resilience to examine how multiple interacting systems of power shape sources and strategies of resilience across the life course. Data from focus groups with Black gay and bisexual older adults provide new insights about resilience—its promises, its limitations, and its application to diverse groups.

This section is organized around four topics. It begins with an overview of the research on Black gay and bisexual older men—underscoring that little is known about what factors support wellness and that challenge the injustice that many Black gay and bisexual older men experience. It next presents the existing research on resilience and social determinants of health and notes a call for more scholarship on resilience that considers complex interactions between individuals and their environments and among underrepresented populations, including Black gay and bisexual older men. It subsequently examines the existing research on resilience among gay and bisexual men. It concludes with the literature examining intersectionality and power, justice, and critical consciousness. This final section notes that intersectionality provides a helpful analytic framework for examining relationships between individual-level experiences and larger systems of power, privilege, and oppression. It thus provides an overview of intersectionality frameworks and connects it to resilience concepts, including “conditions of justice” (or injustice) for resilience and critical consciousness.

### 1.1. Research on Black Gay and Bisexual Older Men

From a life course perspective, Black gay and bisexual older men have faced many types of discrimination over time regarding intersecting positionalities of race, sexual orientation, gender, and age—among other dimensions—across their life courses. Many Black gay and bisexual men also saw their communities devastated by the AIDS epidemic in the 1980s and 1990s, while government officials and religious leaders remained silent [8,9]. These experiences have triggered trauma during subsequent loss in their lives, including the COVID-19 pandemic [10], where many Black gay and bisexual older men experienced multiple risks factors, including death.

Little empirical evidence exists on Black gay and bisexual older men. The existing research on Black gay and bisexual older men underscores the additional challenges that Black gay and bisexual older men face across their life courses, including systematic discrimination [11,12,13], dominant cultural norms and messages about masculinity [14] and race [15], homophobia [16], subtle rules about knowing one’s place within social hierarchies [17], and rejection from family and friends [13,18,19]. These experiences have health consequences and can contribute to health inequities, including poorer physical health, higher rates of social isolation [20], and higher rates of HIV compared to other groups [21,22]. Several studies have found that community connection combats social isolation and has a positive impact on the mental health of Black gay older men [23,24]. However, much less is known about the forces that support wellness and challenge the injustice related to inequities among Black gay and bisexual older men.

### 1.2. Resilience and Social Determinants of Health

Resilience presents a lens for examining mechanisms that support wellness and justice in the context of social determinants of health. Scholars have studied resilience as an individual trait (e.g., grit) [25,26,27], process [6,28], resource [29,30], or outcome [31,32]. Researchers have also examined psychosocial determinants of resilience [33]. Despite these differences, resilience definitions tend to share a core requirement of adversity [19,33,34,35,36]. More recently, scholars have defined resilience as a “collective action to reduce the impact of structural adversity on individuals, institutions, and communities/populations’ ability to thrive” [1]. Resilience may emerge as a process of positive adaptation [34,37] and transformation [38,39] over time. Among resilience research, very few studies have studied the process of resilience with multiple interacting experiences among older adults [11,29,39] or in relation to race [40].

Some scholars have stressed that resilience research tends to glorify resilience, or place too much emphasis on resilience as a personal good [41,42,43]. Davis [41] (p. 5) argues that resilience too often focuses on protective factors among poor people and people of color and instead encourages a more critical reflection on why these groups may be so “imbued with resilience” and who or what should bear the responsibility of the adversity that these groups are encountering.

Other researchers studying resilience have urged researchers to consider complex interactions between individuals and their environments [1,44,45] and stress the need for more research measuring systems and sources of adversity [46,47,48], contexts or “counterspaces” [49,50] that promote resilience, and societal investments in multilevel systems to advance health equity [1]. While terms like collective resilience [38,51,52,53], community resilience [34,53,54,55], organizational resilience [56,57], and system resilience [58] have emerged, most research on resilience focuses on an individual-level analysis [59,60,61] and fails to examine how multiple interacting systems may shape resilience. However, recent definitions of resilience in social determinants of health literature have underscored the importance of considering multilevel systems in the context of resilience and health equity [1].

### 1.3. Resilience Among Gay and Bisexual Men

Despite the sobering research on gay and bisexual older men, some researchers have identified protective factors that may buffer some of the damaging effects of discrimination and adversity experienced by gay and bisexual men. Studies have found that emotional support [62], family support [63,64,65], peer support [20,29,63,64], mastery [62], strong social ties or a sense of belonging to a gay community [66,67], a strong network of social services and support [65], and having a regular physician [68] are beneficial to gay and/or bisexual men’s physical or mental health.

Fewer studies have empirically examined resilience for older gay or bisexual men [11,20,29,63,65,68,69]. Fredriksen-Goldsen et al. [20] identified protective factors such as social support and network size as indicators of resilience among a cross-sectional group of 2439 LGB older adults. Emlet et al. [29] identified social support and community engagement as factors contributing to resilience, as well as previous diagnosis of depression as detracting from resilience, among a subsample of 335 gay and bisexual older men. In a qualitative study of 12 gay older men, Kushner, Neville, and Adams [63] found that strong social supports, such as a partner, friends, and/or family, were critical to facilitating aging for older gay men facing homophobia and heteronormativity. Drawing from 25 interviews with white gay men over age 40, Handlovsky et al. [11] found that participants actively resisted discrimination through three interrelated processes that influenced the development of resilience over their life course: building and sustaining networks, addressing mental health, and advocating for respectful care encounters. Handlovsky et al. [11] advocated for further research that examines processes of resilience development among more diverse groups. In a qualitative study of 16 older men who have sex with men living with HIV/AIDS in Southern Nevada, Ranuschio et al. [65] identified facilitators to promoting resilience, including a strong network of local HIV/AIDS prevention, treatment, and related social services, as well as emotional and mental health support from one’s family of origin or chosen family.

While a larger body of literature examines resilience among people of color [45,70,71], only a small subset of this literature addresses resilience specifically among Black gay or bisexual men [19,40,72,73,74,75,76]. Wilson et al. [40] identified four profiles of resilience among 228 young Black gay and bisexual men in New York City that suggested that self-efficacy and hardiness/adaptive coping possibly provided stronger protective factors compared to social support and that resilience is thus multidimensional. Reed and Lin Miller [19] examined resilience among 23 young Black gay and bisexual men who experienced syndemic conditions (avoiding psychosocial health conditions). They found that men who avoided syndemics had supportive relationships with people who strengthened their sense of identity, provided opportunities to support their communities, and promoted positive norms about health [19]. However, experiences of oppression were connected to shame, social isolation, disconnection, distrust, and expectations of additional marginalization [19]. McNair et al. [75] examined resilience scores among 364 Black MSM in the Deep South and found that resilience may have a protective effect on HIV. Barry et al. [72] examined 322 conversations from 48 Black gay and bisexual MSM who participated in an online mobile phone intervention around HIV prevention and care. They found that participants exchanged social support, engaged in health-promoting cognitive processes like reframing and self-acceptance, enacted healthy behavioral practices around sex-positive norms, and empowered other gay and bisexual youth through role modeling, self-advocacy, and encouragement [72]. Hussen et al. [74] examined qualitative interviews with young Black gay and bisexual MSM living with HIV after a group-level intervention that aimed to develop resilience at the individual, social network, and community levels. They reported that this intervention augmented resilience and social capital among this community, including strengthened bonds and connection strategies, as well as appreciation for intersectional identities [74].

Among the handful of studies examining resilience among Black gay and bisexual men, two studies explicitly applied an intersectional framework. Follins, Walker, and Lewis [73] conducted a critical review of the resilience literature for Black LGBTQIA+ individuals with an intersectional approach. Through this review, they identified factors that may lead to the development of resilience in Black LGBTQIA+ individuals, including support from family, chosen kin (e.g., other Black LGBTQIA+ individuals), and religious communities; a sense of self-efficacy; a sense of self; and active coping strategies [73]. The literature also revealed that many Black LGBTQIA+ individuals gained significant psychological benefits from the integration of multiple positionalities that allowed them to focus on current circumstances and future goals [73]. Quinn et al. [76] also invoked intersectionality frameworks to examine interview data from 44 Black men who have sex with men (MSM) and found pride in intersectional identities, perseverance, community advocacy, and social support. Quinn et al. [76] underscored how Black MSM invoked these assets and resources in response to oppression related to racism and heterosexism. None of these studies, however, explicitly focused on older adults. Given generational differences in access to resources and systems of oppression and support, more research is needed on older adults, particularly LGBTQIA+ older adults of color.

### 1.4. Intersectionality and Power, Justice, and Critical Consciousness

Intersectionality provides an important analytic framework that builds on this emerging literature and helps in understanding the intersection of individual micro-level experiences and interlocking macro systems of power, privilege, and oppression [7,77]. Intersectionality approaches examine impacts of multiple interacting systems associated with such organizing features of society as culture/ethnicity, gender, race, sexual orientation, age, economic class, religion, and dis/ability (also sometimes called “positionalities”) [78,79,80,81]. Incorporating power explicitly helps address concerns that resilience theory places too much onus on the individual and not enough analysis of the systems that require resilience in the first place and provides a helpful framework for this study.

Hill Collins and Bilge [7] identify four domains of power that shape intersections of positionalities: structural, disciplinary, cultural, and interpersonal. Structural power includes how social institutions are organized to reproduce subordination [82]. Disciplinary power includes how governments, bureaucracies, and other actors regulate thought and behavior through subtle rules, practices, and social processes [82,83]. Cultural power encompasses the creation, perpetuation, and values attached to meanings, interpretations, and ideologies [7]. Interpersonal power involves everyday interactions among people [7]. For purposes of this article, we add a fifth domain: intrapersonal internalized oppression and domination [84]. These domains of power are mutually constructed and thus do not exist in individual silos [7]. They provide important theoretical tools to explain sources of inequity as well as components of different social contexts, including potential sources of and strategies for building resilience.

Prilleltensky [85] (p. 15) argues that one’s ability to successfully cope with stressful life events depends on “the balance of power between protective and risk factors.” Intersectionality frameworks would add that this balance of power is less a system of additions and subtractions but instead more of a mutually constructed multilevel system that interacts with and on one another to create different circumstances of privilege and oppression. Prilleltensky [85] (p. 8) further contends that optimal conditions of justice—which involve striving to achieve full potential and being fulfilled—promote thriving [85] (p. 12). Suboptimal conditions of justice—which involve a lack of resources or assault on one’s system—produce coping responses such as resilience [85].

According to Prilleltensky [85], conditions of injustice may prompt individuals to confront a system by engaging in three psychosocial responses: critical experiences that lead to revelations about injustice; critical consciousness that change can happen; and critical action for social change. Building on Freire [86,87], other scholars define critical consciousness as encompassing all three of these responses, including (1) critical reflection (awareness of how social, economic, and political conditions stifle opportunity and reproduce systemic injustices); (2) sociopolitical efficacy (perception of one’s ability to act and change these conditions); and (3) critical action (the extent to which individuals participate in individual or collective action) [88,89]. Collins & Bilge [7] also underscore the importance of critical consciousness as a tool for analyzing injustice and taking action but add that individuals benefit from a critical consciousness of how intersecting social inequalities and positionalities shape their circumstances and opportunities for change.

Invoking intersectional frameworks of power, justice, and critical consciousness may help explain the processes and strategies that older Black gay and bisexual men employ to build resilience and move toward thriving. This study aims to contribute a deeper understanding of how gay and bisexual Black older men navigate complex systems of power and particularly how intersecting systems of power shape the sources of and strategies for developing resilience and thriving. This project thus asks the following research questions: How do intersecting structural, cultural, disciplinary, and interpersonal systems of power shape the sources of resilience among Black gay and bisexual older men? How do Black gay and bisexual men build resilience and move toward thriving amidst these systems of power across their life courses? We use the terms Black, gay, and bisexual to reflect the terms most frequently employed by the participants in the focus groups.

## 2. Materials and Methods

### 2.1. Data Collection

We used purposive sampling [90,91] to conduct two intracategorical focus groups [92] with Black gay and bisexual older men (*n* = 17). Focus groups are especially beneficial for exploring complex phenomena and needs among minoritized communities [93] and when participants have something in common and share a stake in the issue being discussed [94]. Focus groups allow participants to bounce off ideas, diversify opinions, and flesh out issues more comprehensively [95]. The intracategorical design focuses on the complexity of experience *within* a particular social position, positionality, or intersection [78,92,96] to explore nuances in experiences among Black gay and bisexual older men who were diverse in other ways, including education, religion, and disability. This approach allows for an analysis on how people make sense of and respond to the positionalities that shape their lived experiences. Positionalities reflect one’s positions of power in relation to others in various social, political, and economic structures, as well as cultural contexts and interpersonal dynamics [97,98,99,100]. Intracategorical focus groups allow for a more nuanced and in-depth intersectional examination of differences (and commonalities) among a group with shared intersectional positionalities [78,101]. Here, everyone in the focus groups identified as Black, older, and gay or bisexual, and these focus groups thus reflect what we call a “multi-intracategorical” approach because all participants shared four common intracategorical positionalities regarding gender (male), race (Black), sexual orientation (gay/bisexual), and age (older adult). This hyper-focused approach allowed for a more targeted analysis among a group whose intersecting positionalities are rarely examined or included in research: Black gay and bisexual older men.

Participants from these intracategorical focus groups were part of a larger study (*n* = 31 focus groups) using a transformative framework [102] with a sequential-methods design [103] and social justice goals. A major objective for the larger project was to systematically explore how to operationalize critical intersectionality frameworks [7,82,101,104] in research in different contexts with different populations (e.g., LGBTQIA+ older adults, MENA youth and adults, adults with disabilities, Black mothers, etc.).

Limited empirical research on LGBTQIA+ older adults of color motivated this subproject to focus on this subsample as well as a desire to identify sources and processes of resilience amidst intersecting systems of adversity. This subsample also serves as an example of an extreme case sampling strategy, which selects participants who represent a highly specialized or particularized group to provide a richer and more in-depth understanding of a phenomenon [90,91]. Given our interest in processes of resilience and thriving, we wanted to focus on an LGBTQIA+ aging community that had likely experienced historical trauma and cumulative discrimination because of intersecting positionalities but was also actively engaged in their communities. Black gay and bisexual older adults have experienced intersecting systems of discrimination because of their race, gender, sexual orientation, and more recently older age, among other dimensions. Their cumulative experiences of intersecting discrimination can also help reveal strategies of resilience that may be less visible within other communities.

To identify individuals engaged in local LGBTQIA+ communities, we partnered with a community group that focuses on providing monthly support and discussion for Black gay and bisexual older men in a large urban setting to recruit 17 people to participate in our research project through an extended monthly group meeting. Given that the target population for this study is vastly underrepresented in research, the research team leveraged existing community networks and community trust built from decades of research and community engagement with community participants from this support group in this particular locale. Our mean age was 55, but participants ranged in age from 39 to 74.

While most aging-related research begins at age 50, we incorporated one participant who was as young as 39, given research on accelerated aging and cumulative impacts of stress and discrimination for multiply minoritized populations [105,106,107]. The participant who was 39 also was a HIV long-term survivor, which has been associated with accelerated development of disability and chronic diseases, which can shorten one’s lifespan considerably [108,109]. For example, one study found that the prevalence of multiple noninfectious comorbidities of people 40 and over with HIV was similar to adults without HIV who were 10 years older [110]. Another study found that the prevalence of plaque in people living with HIV was similar to those who were 10 years older and can accelerate cardiovascular disease at a younger age for people living with HIV [111]. Another study found that age-related comorbidities can occur 16 years earlier among insured individuals living with HIV than those without HIV [112]. Given this research, we believed an intersectional methodological framework should incorporate this participant into this study. See Table 1 (Participants’ sample description) for additional information about the participants.

We learned in earlier work from the larger project that it is important to provide some core definitions and allow participants to individually explore some core concepts, positionalities, dimensions, and contexts in their lives that were expected to be relevant in the focus groups. Before conducting focus groups, we thus provided a brief questionnaire for participants to complete. Participants completed this questionnaire in a large group after all questions were answered about goals, confidentiality, and general norms and procedures of the study. The questionnaire asked about the language they used to describe their gender and sexuality and other positionalities (e.g., race/ethnicity, economic and dis/ability status, and health), as well as some current social contexts (partner status, people living with, giving and receiving caregiving). These were followed by a series of scales exploring experiences of everyday discrimination (implicit and explicit biases), mental and physical health, and vigilance to prepare for discrimination. After each set of scales, participants were asked what they thought about the reasons for the items they had checked; these included many positionalities or other characteristics associated with positionalities or forms of discrimination.

After completing the questionnaire, the participants were divided into two groups in two different rooms, each with a facilitator, co-facilitator, note-taker, and two recording devices. Before beginning the focus group questions, facilitators conducted a structured experiential activity in which participants identified some of their core positionalities and then engaged in a self-assessment where they reflected on whether and how much of each of these were relevant in various contexts in their lives. This experiential activity also continued to introduce concepts relating to positionalities to focus group participants before the focus group discussion began.

Pilot work taught us that these “priming activities” (e.g., the questionnaire and self-assessment that could be shared at the beginning of the group), which provided an introduction to key concepts and an opportunity to consider their own multiple positionalities and current and past experiences, was essential for people to identify and contribute their own experiences and not just be influenced by those most active in the focus groups [63]. For this paper, we focus primarily on responses within the focus groups, using questionnaire data primarily for describing the sample.

We used focus groups to elicit interactive discussions that would have been unlikely to surface in individual interviews [94]. Drawing from focus group research design [94,113], we conducted two focus groups with no more than ten people because (1) our group was so particularized, and we wanted to gain a deeper understanding about their individual and collective experiences [94], and (2) we wanted to provide a space where participants could share and compare [114] with a sufficient number of participants to build “safety in numbers” [113] (p. 42). The focus group protocol included topics such as self-perception about positionalities, reports of how others react to them, how different social contexts influence these, and how these have changed over time.

### 2.2. Data Analysis

We used multiple forms of triangulation in data analyses [multiple investigators, several analytic frameworks, working across methods, using various types of tools (Dedoose, Excel, memo writing), and different kinds of coding]. After transcribing the focus groups, the research team incorporated a hybrid approach to thematic coding [115,116] in Dedoose [117] that used inductive and deductive approaches [118,119]. First, the team conducted open coding to develop a thematic codebook that we used to carry out deductive coding. The codebook had nine core parent codes informed by research memos and team discussions after the focus groups were completed (i.e., domains of power, HIV, church, coming out, hypocrisy, money, rejection, resilience, support). Additional team discussions led to a subsequent focus on resilience and a second round of thematic coding that included a theory-informed analysis [120]—informed by empirical, theoretical, and conceptual literature on resilience (e.g., adversity, support, community, systems, different levels of wellness, and psychosocial processes). The authors regularly met and exchanged documents discussing emerging themes in the data, which added rigor to this iterative and inductive process [121]. We used analytic memo writing [122] to draw conceptual connections between coded data and emerging conceptual frameworks.

The research team represented many positionalities and employed self-reflexivity throughout the research project. Reflexivity is a process of researchers’ looking inward, questioning one’s taken-for-granted assumptions, potential biases or standpoint(s), and how our roles, experiences, and perspectives shape knowledge production [123,124]. The research team included people who reflected shared positionalities regarding the participants regarding race, gender, sexual orientation, or age. We met often to discuss the analysis process, including how our positionalities may shape the research process, which helped us interrogate our positionalities and thoughtfully engage with the data and research process. For example, we noted how different researchers foregrounded different findings based on shared (or dissimilar) positionalities with the participants. These discussions helped triangulate the data and produce a more robust data analysis process. These conversations and reflections were captured through research team discussions and memos.

## 3. Findings

All names identified in the findings below are pseudonyms. Several themes emerged from interpretive analyses of focus group data that revealed both positive and negative sources of strength from multiple levels and domains of power. These themes highlight how intersecting structural, cultural, disciplinary, and interpersonal systems of power shape sources of resilience among Black gay and bisexual older men and how Black gay and bisexual men build resilience and move toward thriving amidst these systems across their life courses. Different times and environments created varying conditions that participants had to navigate: periods of suffering for many, examples of feeling vulnerable, developing critical consciousness and engaging in advocacy, and confronting unfair conditions. Interacting systems of power shaped both positive and negative sources of resilience. Participants gained strength from positive support networks, including biological family and families of choice. However, they also gained strength from sources of deep conflict at all levels of power that required participants to adapt as gay or bisexual Black men. Some participants also had HIV. They described many negative experiences with discrimination in housing, employment, education, and religion (structural); cultural biases that compromised their safety (cultural); subtle and rigid rules regarding gender, sexuality, and family (disciplinary); and stigmatizing interpersonal exchanges (interpersonal). Participants explained how these negative experiences produced complex individual strategies of critical consciousness (intrapersonal) that helped build resilience and helped them thrive at various times, including naming external ignorance, acknowledging common struggles, and reconciling contradictions.

### 3.1. Sources of Resilience

#### 3.1.1. Positive Support Networks

*Biological Family Support*: Many participants described the importance of biological family support in buffering intersecting oppressive structures. For example, Clarence described how good family ties provided critical support while coming of age during a segregated Jim Crow era when being gay and Black were deeply stigmatizing social positions.

When I was comin’ up, my parents were from the South. As a kid, they said that I’ma show you where you came from and it was still segregation. I remember eatin’ in the back of the restaurant, downstairs, segregatin’ the phones, the busses. Then, when I got of age, when I discovered my sexuality, I had good family ties behind so they embraced it. My name was Clarence Smith and I was a person.

Clarence expressed deep pride in his family name and his value as a person. While dominant structures of segregation and cultural norms of racism and homophobia prevailed among structural, cultural, disciplinary, and interpersonal systems, Clarence described a sense of worth that he attributed several times to having “good family ties.” Family support also rendered him visible amidst a larger society that devalued his existence.

Intersecting structures of oppression also facilitated biological family support for other participants. LeRoy explained that when he shared his sexual orientation with his mother, she responded by asking “what did I want, a medal?” When he expressed his concerns that she would be upset, she quickly brushed them away and treated his sexual orientation as a nonissue, despite living in an era when being gay was not particularly accepted. During other parts of the focus group, LeRoy described some of the challenges that his family—including his mother—experienced. His family was economically struggling in a social structure that was unwelcoming to African Americans. Yet LeRoy also came of age when significant social, political, and economic transformations were happening all around him. While being gay was still very stigmatizing, it is possible that these massive changes and intersecting oppressions blunted the significance for his mother, who was just trying to survive day-to-day in a rapidly changing world with rampant explicit and subtle discrimination.

Family support was sometimes an evolving interaction. Malik described a process of evolving family support and resilience after he was diagnosed with HIV.

When I got HIV, that was the only thing they could understand when I went to go shake their hand and they’d go like this, but what I was proud of as far as my family and people that I knew, they took time to study. It said you cannot catch it from a handshake, and so it went from this to a hand or a hug. I was like okay, they took time to do that. I’m really glad they did.

This experience underscores the complex intersecting domains of cultural, structural, disciplinary, interpersonal, and intrapersonal power. Malik was diagnosed with HIV during a time when it was still deeply stigmatizing, and massive misinformation existed in the public about how one contracted it. Structural systems of discrimination and cultural values and meanings attached to HIV produced deep stigma about HIV and how individuals would interact with him. Along with white gay men, the AIDS epidemic also ravaged many Black communities (gay, transgender, and heterosexual). Malik’s family was likely conflicted by their own fears, ignorance, and love for Malik. Malik describes an intrapersonal sense of power and positive self-worth after his family accepted him and took steps to learn more about HIV.

*Support from Families of Choice*: Participants also described a robust support network of nonbiological family, including “houses” (nonbiological families of Black, Latine, LGBTQIA+, and gender nonconforming youth that aim to create a space of acceptance, nurturance, and safety) and support groups. William acknowledges that “[i]n the gay community, it’s a system of houses, right? These places where they have—those outcasts commune together.”

Several of the participants agreed that houses served important roles in their early lives. However, as they have aged, they have found new sources of support from families of choice, including support groups with varying levels of formality. Ron described how Urban Gay Elders (a support group for older Black gay and bisexual men) provided “[t]he ability to be able to converse freely.” James added that “[t]hings I may not say in some other groups, I can say” in this support group. John acknowledged that “[t]he building—the people in the building—are acceptable to us and what we do. All of that makes a difference.” For Ron, James, and John, nonbiological family continues to provide instrumental support to buffer oppressive systems that mark them as “outcasts” and hinder their ability to feel free to authentically represent themselves in public. As youth and young men, they relied more on “houses” that did not necessarily provide physical space but provided emotional support by redefining interpersonal and cultural domains of power and justice. As they aged, they transitioned to more formal support groups that were housed in physical structures of support. Despite increasing challenges with mobility and aging that some participants mentioned throughout the focus groups, they still drew support from attending physical meetings in physical spaces and structures that provided support. Being visibly accepted in the building also suggests the importance of cultural power and meanings, interpretations, and values attached to them as publicly out older Black gay and bisexual men. John’s comments also underscore how inviting spaces and allies can facilitate necessary structural support for families of choice to blossom and overcome adversity.

#### 3.1.2. Intersecting Barriers and Conflict

Positive support networks were not the only source of strength and resilience for participants. Many participants also described negative experiences as a source for resilience. These negative experiences reflected their intersecting positionalities as Black gay men within a network of relationships, structures, cultural constructions, social processes, and systems of inequality. For example, Paul described how visible markers of stigma regarding gender and race require strength. Being a Black man required Paul to develop a robust inner strength to overcome the stigma ascribed to these visible positionalities. Because his gender and race were very visible, he was subject to both overt and subtle forms of discrimination and harassment at intersecting levels of oppression. In addition to structural discrimination, he also encountered cultural stereotypes such as being incompetent, violent, or lazy that were tied to his intersecting racial and gender positionalities, which also affected how people interacted with him. His sexual orientation was less visible. Elsewhere in the focus group, Paul acknowledged himself as a gay man and noted on his questionnaire that his sexual orientation prompts him to be careful about his appearance, carefully watch what he says and how he says it, and prompts him to avoid certain situations and places. However, Paul did not list his sexual orientation as one of his strongest sources of strength, nor did he identify it in conjunction with race and gender. While Paul acknowledged the deep stigma attached to being gay, his sexual orientation was less visible and may have required less labor to overcome stigma compared to more visibly stigmatizing positionalities such as being a Black man.

While race and gender were most visibly stigmatizing for Paul, another participant, Morgan, described how visibly displaying a particular skill (sewing) prompted family members to reject him because of this skill’s cultural association with being gay.

I’m teaching my nephew. He wanted to know how to sew. I’m setting up curriculum, teaching him to sew. He’s motivated. His father—my nephew didn’t come back. I’m like, how come? My dad says that this is gonna cause me to be gay. *What?!* How is teaching you a skill—and I’m like, your father doesn’t get—so I talked to my sister. She doesn’t stand up. I’m like, really? How ignorant can you both be? Then ask your daddy, who does he buy his clothes from? Nobody’s asking him to go to bed.

Morgan’s logical appeal to his sister failed to override her husband and her concerns that having their son learn this skill would prompt him to be gay. Cultural values about masculinity and sexuality dominated their view of what practical skills Morgan could teach their son. Morgan’s family treated his sexual orientation like a contagion, and sewing was the vehicle for transmission. Morgan also has HIV, yet he made no mention of his family’s concern about him transmitting HIV to his nephew, despite the stigma he faces in other contexts because of his HIV status. Nevertheless, Morgan expressed pride in his sewing skills and recognized his family’s response as “ignorant.” Ultimately, Morgan’s pride seemed to transcend his family’s rejection and reveals how resilience can emerge among family conflict.

Another participant, LeRoy, described how living in an impoverished neighborhood with high crime gave him strength and survival skills. He noted that living in this neighborhood required that he render his sexual orientation invisible. He could not be safely visibly gay. He begrudgingly added that “what don’t kill you makes you stronger.” LeRoy experienced intersecting structural discrimination as a young, poor, youth of color living in a segregated, impoverished neighborhood with significant violence. Cultural constructions of gender, race, and sexual orientation also created subtle but strong rules that he could not stray from racialized norms of masculinity that were key for his survival. LeRoy shared how these intersecting systems of oppression shaped sources of resilience in ways that subsequently helped him combat other forms of discrimination, including homophobia.

#### 3.1.3. Religion

Religion represented a space of both positive support networks and conflict for participants that could serve as a complex source for resilience. For example, James described how a personal relationship with God gave him the strength to overcome many obstacles by showing “what we all are capable of doing, what we can endure, what we can achieve.” James describes God as a mentor and guardian who has helped him tackle barriers in his life, given him strength to accomplish new goals, and helped him engage in more open and truthful conversations. James placed deep value on honesty. He previously noted in his focus group that he could not be open with his sexual orientation as a young adult. He also reported in his questionnaire that he now feels at ease with himself and never feels the need to be careful about what he says or wears because of his sexual orientation. James feels comfortable being a Black gay man and seems to attribute this comfort, in part, with his relationship to God. God served as a buffer for some of the structural and cultural forms of oppression he experienced as Black gay man, and he developed a source of interpersonal power with God that served as a source of resilience.

Roger described a more nuanced and compartmentalized approach to religion that draws from intersecting systems of power.

When you go in there, you go for spiritual growth. You’re not goin’ for that preacher up there or whoever else lookin’ at you. They come up with that scripture, Leviticus, you need to understand what Leviticus is talkin’ about and understand the truth between you and God who created you, not who’s preaching and tellin’ you what you are.

While Roger recognizes the existence of his preacher’s homophobia as powerful, he refuses to let these comments keep him from church and thus relies on his own interpersonal relationship with God. He draws from his own intrapersonal expertise of the Bible that grew from years of church participation. The cultural meanings and values attached to belonging to a church ultimately outweigh the stigma he faces as a Black gay man there. His experience reflects complex sources of resilience stemming from positive and negative experiences stemming from intersecting structural, cultural, disciplinary, interpersonal, and intrapersonal domains of power.

In contrast to James and Roger’s experiences, several participants expressed deep disappointment and rejection from their churches that ultimately helped build resilience as Black gay men. They admonished preachers who referred to LGBTQIA+ people as “abominations” and explained that they either temporarily left unwelcoming churches or considered themselves nonreligious. For these participants, religion was a source of structural and cultural oppression that resulted in stigmatizing interpersonal interactions and internalized stigma. However, the hypocrisy exhibited by their church leaders and fellow congregants also revealed the fragility of these systems of oppression in ways that gave them strength. Systems so imbued with contradiction helped them realize that they did not need to seek support from their churches but instead could develop new support networks that valued them holistically as Black gay men.

### 3.2. Building Resilience and Thriving Through Critical Consciousness

Three strategies emerged in how participants coped with negative experiences: (1) naming external ignorance; (2) acknowledging common struggles; and (3) reconciling contradictions. These strategies involved various levels of critical consciousness as participants aged. Critical consciousness, thus, helped Black gay and bisexual men build resilience and move toward thriving amidst systems of power across their life courses.

#### 3.2.1. Naming External Ignorance: Stuck on Stupid

When faced with various forms of oppression or discrimination, several participants employed a strategy in which they named the oppressive or discriminatory act as “ignorant” or “stupid.” One participant, Chris, described family who were “stuck on stupid” because of how they responded to other family members with HIV. Roger added that people who are “stuck in stupid mode” are often the “folks that are doin’ the judgin’ [and] are some of the worst offenders.” Ron subsequently applied the term “stuck on stupid” to preachers who referred to LGBTQIA+ people as an “abomination.”

Naming the oppression or discrimination seemed to help some participants cope with external stressors that caused them pain. It required some sense of critical reflection about how social, economic, and political conditions were stifling opportunity and perpetuating injustice, a key component of critical consciousness. Naming these sources of pain as coming from sources outside of themselves allows them not to blame themselves (internalizing oppression and absorbing the stigma), which is one of the more debilitating consequences of oppression. This then enables participants to reclaim power and exhibit resilience that transcended the offending actions. Many participants who named external ignorance also expressed sociopolitical efficacy, or a perception that they had the power to act or change these conditions through a variety of ways. Many participants who described oppressive churches engaged in critical action by changing churches, abandoning religion, developing a very personal relationship with God, engaging in personal confrontations with individual spiritual leaders or fellow congregants, or working for other systematic changes within their churches. Other participants who described “ignorant” family members or colleagues as “stuck on stupid” did not actively engage in sociopolitical efficacy or critical action, but several participants described sociopolitical efficacy and critical action when detailing conversations with family and colleagues to change their “ignorant” positions. Regardless of whether or not they engaged in actions to change their environments or the success of these efforts, participants nonetheless exhibited critical consciousness that helped them to avoid absorbing stigma (internalizing the oppression) of being Black gay men in these spaces.

#### 3.2.2. Acknowledging Common Struggles

Many of the participants shared common struggles of discrimination. Acknowledging these shared experiences helped explain another strategy that some participants employed to build resilience and thrive. For example, Morgan described how recognizing shared struggles with other low-income African Americans helped build his resilience as his neighborhood became increasingly white and middle class through gentrification.

It’s changed the way I perceive myself, my ability. It’s made me a little bit more resilient. I realize that I can do a lot more than I anticipate I could and I can do a lot more for the good. This change has been a learning curve. I’ve had to learn how to deal with less and how to be acceptable with dealing with less and that wasn’t easy because it was never questioned before. Part of that is knowing—understanding that it finally hit me. I’m a Black male and I’m finding that I’m struggling the same way that other Black males were, whereas before I was in a position where I didn’t have to be a part of the struggle. It’s changed the way I perceive things.

Morgan’s experience with gentrification sharpened his awareness about his positionalities, particularly regarding race and class. Morgan engaged in critical reflection when he realized that he was experiencing a shared structural and cultural shift in his neighborhood with other low-income African Americans that “made [him] a little bit more resilient.” His comments also reveal a sense that he has the ability to create some sort of change and engagement in critical action that was transformational. By aligning himself with other African Americans in his gentrifying neighborhood, Morgan moved into a role as an active member “of the struggle.”

The informal support groups that many participants mentioned earlier also reflect resilience born from shared struggles. For example, Urban Gay Elders was formed to support Black gay older men who shared common experiences of exclusion and stigma based on their race, sexual orientation, and gender as they aged. Roger noted another support group of Black “same-gender attraction” men that formed after a dearth of support for the many Black men who were dying of AIDS in the 1980s. This group emphasizes “commonality over difference” and encourages participants to embrace their “individual and unique” gifts. Both of these support groups emphasize the emergence of critical reflection, sociopolitical efficacy, and critical action that grew out of necessity and response to shared traumas as Black gay and bisexual men.

#### 3.2.3. Doing the Journey: Reconciling Contradictions

A third strategy that participants employed to build resilience and thrive involved “doing the journey.” This includes evolving decisions and personal reflection over a sustained period of time. Roger more specifically invoked this strategy when explaining how he drew support from a recent support group that encouraged participants to “do the journey” on navigating conflicts between spirituality and sexuality. Given the variety of responses participants had to religion, “doing the journey” seems especially fitting. Roger recognizes that this journey may itself be littered with challenges. However, it is premised on the possibility of peace—of reconciling sources of conflict in one’s life. For Roger, this journey involved reconciling conflicts between his sexual orientation and spirituality.

For other participants, like John, the “journey” can involve self-care while aging. John agreed to help his nephew by letting him live with him for free while working a low-wage job. John described the conflict he felt with supporting his family and tending to his own needs as his nephew continued to challenge his boundaries. Ultimately, John evicted his nephew after he failed to treat his home or him with the respect he thought he deserved. John’s decades of life experience and position as an aging adult shaped his outlook and expectation for respect. When his nephew failed to meet that expectation, John evicted him. He was conflicted by a critical reflection of the structural barriers that his nephew faced and his own material needs as an aging Black gay man. He expressed an awareness and engaged in action to create justice for himself but acknowledged that this action occurred at the expense of his nephew’s own well-being.

Other journeys for self-care include transitioning support networks as needed, including moving from “houses” to informal and formal support groups as described by participants above. These journeys require deep critical reflection to negotiate emerging conflicts that stem from systemic injustices. By engaging in the journey, participants also recognize their own ability to act and change their conditions. Finally, the outcome of their journey often results in an individual or critical action that aims to address an injustice.

## 4. Discussion

Intersectionality provides a useful lens to understand the sources and strategies of surviving and thriving, including resilience among Black gay and bisexual older men in the context of social determinants of health. These experiences were shaped by multiple intersecting positionalities regarding their race, gender, sexual orientation, health status, and age, among others. By positioning these experiences within various domains of power [7], we also can see how participants’ experiences and responses are shaped by multiple levels of power that interact with one another in complex ways.

**Figure 1:**
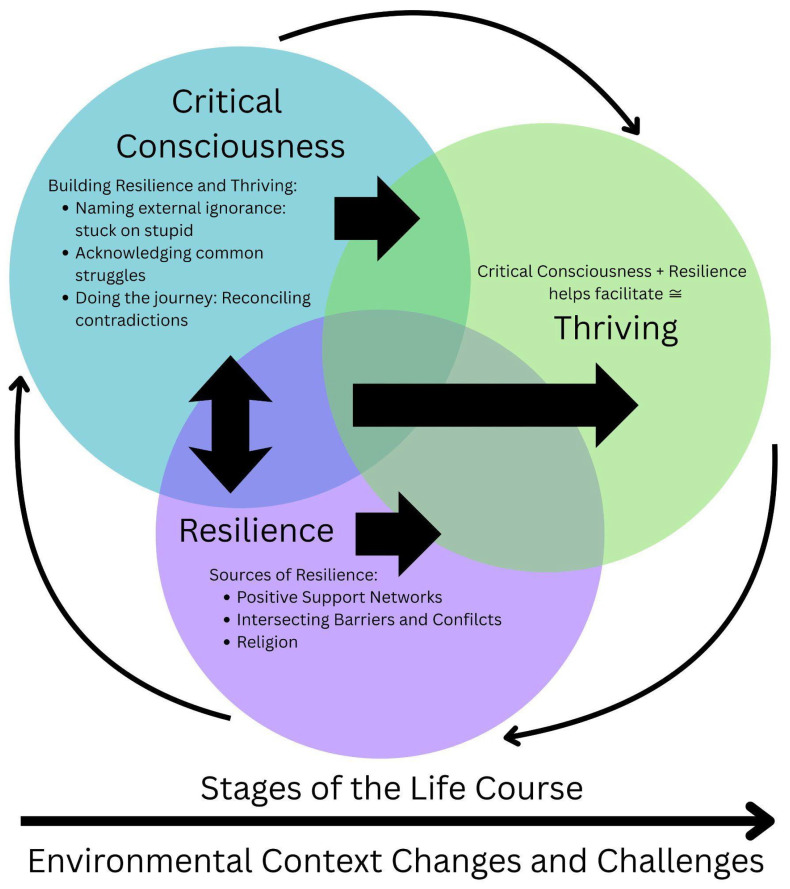
Conceptual framework connecting critical consciousness, resilience, and thriving.

We identified a wide range of positive and negative experiences that helped shape sources of and strategies for building resilience and thriving through critical consciousness as Black gay and bisexual men navigated their life courses and were also shaped by changing contexts over time. See Figure 1 (Conceptual framework connecting critical consciousness, resilience, and thriving).

Figure 1 shows how critical consciousness presents a potential pathway for resilience by providing a framework for understanding and addressing systemic oppression. Resilience, in turn, allows individuals to navigate challenges related to critical consciousness work, such as potential negative impacts on mental health, and to persevere in their efforts to create positive change. When individuals develop both critical consciousness and resilience, they may be better positioned to thrive, experiencing personal growth and contributing to positive social change. In essence, critical consciousness provides the lens, resilience provides the strength, and together they can lead to thriving, particularly for those facing systemic barriers.

While the data revealed interesting insights about the evolution of family support, it is hardly surprising that participants drew strength from positive support networks, especially given research on the importance of emotional [62], family [63,64,65], and peer support [20,29,63,64]. However, participants also drew strength from *negative* experiences. This finding extends previous resilience research that examines protective measures as facilitating resilience and risk factors hindering resilience [11,19,20,29,62], including in the context of social determinants of health [1]. Here, we find evidence that suboptimal conditions of justice facilitated resilience (as Prilleltensky [85] proposes). For example, visible markers of stigma (e.g., race and gender) may have facilitated resilience for several participants (including through community support networks), although at least one participant (LeRoy) also suggested that he drew strength from his emotional and physical labor in keeping his sexual orientation invisible for safety reasons. Religion also served as a conflicting source of resilience. Some participants described the direct support that it provided, whereas others described a more complex relationship with religion. Some participants seemed to recognize the resilience they gained on their journey in negotiating conflicts with religion. Given the complex intersecting systems of power and oppression that the participants experienced across their lives, none of them had “optimal conditions of justice” that would allow them to thrive, as suggested in the framework for wellness that Prilleltensky [85] proposes. However, ultimately, the cumulated experiences of building resilience seemed not only to help some participants survive but also helped them thrive as they aged as individuals and in communities. Given growing efforts to dismantle supports for LGBTQIA+ communities, communities of color, and older adults in the United States and beyond, this research provides some guidance to practitioners, researchers, and policymakers on potential avenues for mental health support amidst these growing challenges.

This study has several limitations. While generalizability is not the focus of qualitative research, we acknowledge that we cannot generalize these findings much beyond the experiences of this sample. The sample size is small, and because participants were recruited through a support group for Black gay and bisexual older adults, this study was limited to studying processes of resilience and thriving among Black gay and bisexual men who had already developed some level of critical consciousness. This could have overemphasized resilience in a sample that already has access to support structures, including the support group Urban Gay Elders. It is possible that the community support group itself fostered what Thimm-Kaiser and colleagues [1] (p. 489) define as resilience through “collective action to reduce the impact of structural adversity.”

Despite these limitations, members of these groups reported origins in different geographic regions, as they or their parents migrated seeking work or better circumstances from other parts of the United States and the Caribbean. They also included members from different generations (and therefore different formative environments) among those considered “older adults.” In addition, some were newer to the support group and were actively seeking mentorship by those who demonstrated more evidence of thriving and different stages of critical consciousness. The findings underscore the multiple types of power and adversity that must be navigated and the diverse circumstances that can provide opportunities for growth and well-being. In particular, these findings emphasize the significance of considering how communities can leverage experiences of adversity as protective measures against future measures of adversity—even if these experiences are still unwelcome. This point may be particularly useful for scholars studying aging in understanding how decades of encountering multiple forms of oppression may better prepare certain groups for tackling new challenges associated with aging.

Overwhelmingly, resilience research has found that support—in its many forms—seems to help in overcoming adversity [20,29,62,63,64,65,67]. The findings here underscore the importance of interventions that build critical consciousness at multiple levels and acknowledge the role that intersectionality frameworks can play in building resilience and helping individuals thrive, even when they lack optimal conditions of justice.

## 5. Conclusions

This study has important implications for research and practice. First, it contributes to the current resilience literature by incorporating a nuanced examination of interacting systems of power. Scholars have called for more research on the role of systems in resilience [46,47] and the complex interactions between individuals and their environment [44]. Using intersectionality as an analytical framework, this study provides insights about how multiple interacting systems of power and justice can shape sources of and strategies for resilience and thriving. Second, this study contributes empirical research on a highly understudied population: Black gay and bisexual older adult men. While the body of resilience literature is burgeoning, it mostly involves white participants. Researchers have called for more research on diverse populations, including in and beyond the resilience literature. However, the insights from these Black gay and bisexual older men also reveal important underlying processes of resilience across the life course that may be helpful in future research for other populations. Finally, this study unpacks complexities regarding critical consciousness and resilience and provides data that could guide mental health practitioners, community-based organizations, and policymakers about how to support Black gay and bisexual older men in developing critical consciousness and resilience that helps them thrive, particularly amidst intersecting systems of oppression. The importance of the support group and other experiences they report suggests many avenues for increasing critical consciousness and resilience to increase the likelihood of conditions for thriving. This research also underscores the importance of building more funding and supports for multilevel interventions that target mental health, including programs and policies that support building resilience and critical consciousness by leveraging multilevel avenues for support.

## Figures and Tables

**Table 1:** Participants’ sample description.

Positionality *	Frequency
Race and Ethnicity	Black or African American (16); African (1); Caucasian/White (2); Native American, American Indian, Alaskan, Native, First Nations (2); Multiracial (2); Blackfoot (1); French (1)
Gender	Male (17), Two-Spirit (1), Androgynous (1)
Sexual Orientation	Gay (14), Bisexual (1), Asexual (1), Same gender attraction (1), Enjoy men (1), Attracted to men (gay), like what I like! (1)
Age	Mean (55.35) Standard Deviation (9.151) Range (39–74)
Religion	None (5), Catholic (2), Islam (1), Christian (3), Buddhism (1), Protestant (5), Jehovah’s Witness (1), Episcopalian (1), Baptist (4), “Very Religious/Spiritual (Undefined)” (1), Nondenominational (1)
Highest Educational Attainment	High School Diploma/G.E.D. (8), Associate’s Degree/Some College (3), Bachelor’s Degree (4), Master’s Degree (4)

* Note: Some participants indicated more than one category on many positionalities. Table 1 also reflects a narrative participant description to better capture the complexity of participants’ intersecting positionalities, as self-reported from participants through multiple “check all that apply” and write-in categories from a demographic survey. While unconventional, we believe it aligns with critical intersectionality scholarship [7,82,101,104] by presenting the complexity of intersecting positionalities as participants described themselves, which is sometimes harder to capture in separate table cells in a traditional table.

## Data Availability

Due to IRB and ethical restrictions with the community partners and participants, we are currently unable to share the raw qualitative data. However, examples of the coding process and focus group protocol are available upon request. This study was not preregistered.
